# Metformin: decelerates biomarkers of aging clocks

**DOI:** 10.1038/s41392-024-02046-1

**Published:** 2024-11-13

**Authors:** Ram Abou Zaki, Assam El-Osta

**Affiliations:** 1 Baker Heart and Diabetes Institute, Epigenetics in Human Health and Disease Program, Melbourne, Vic Australia; 2 Baker Department of Cardiometabolic Health, The University of Melbourne, Parkville, Vic Australia; 3 School of Translational Medicine, Department of Diabetes, Monash University, Melbourne, Vic Australia; 4 Department of Medicine and Therapeutics, The Chinese University of Hong Kong (CUHK), Hong Kong SAR, China

**Keywords:** Translational research, Regeneration and repair in the nervous system

A recent study published in *Cell* shows the widely known and used antidiabetic drug metformin, has geroprotective benefits in non-human male primates.^1^ Metformin is a derivative of biguanide, synthesized in the 1920s and quickly became the first line off-label treatment for type 2 diabetes due to its efficacy in lowering plasma glucose levels.

On 29 December 1994, metformin was approved by the U.S. Food and Drug Administration (FDA) for diabetes treatment. The recent study by Yang and colleagues has assessed the long-term influence of metformin therapy in delaying molecular aging clocks in healthy male cynomolgus monkeys aged 13–16 years. This is the equivalent to 40–50 years in humans. Not only was aging assessed in terms of the DNA methylome, the transcriptome and proteome were also evaluated including the metabolome. Monkeys were randomly assigned to a metformin- (20 mg/kg daily) or a control-group. Additional groups included in the study were younger monkeys aged 3 to 5 years and middle-aged monkeys between 10–12 years of age. All groups were maintained under identical care conditions for 3.3 years, approximately equivalent to 10 human years.

The study assessed gene expression of 79 tissues/organs using RNA-seq and found metformin treatment inhibited age-related pathways, such as inflammation, fibrosis, cell death, and the formation of reactive oxygen species. Indeed, metformin reactivated pathways that were normally repressed with aging, such as lipid metabolism, Wnt signaling, and mechanisms of DNA repair.

All this good rejuvenating news isn’t surprising, infact it vindicates the importance of the cell cycle, which plays a role in the aging process. As we know, a decline in p21 positive cells is a hallmark of attenuated age-related fibrotic changes and lipid peroxidation and the authors observed this in the heart, kidneys, skin, lungs, liver, and stomach of metformin treated animals. This compelling observation was critical because p21 suppresses the expression of genes involved in cell cycle arrest and this directly influences aging.^2^

It isn’t easy to see how metformin could decelerate aging, so the study authors developed the Elastic Net regression model to calculate aging rescue scores by integrating epigenomics, transcriptomics, proteomics and metabolomics data sets. Administration of metformin decreased the protein age by an average of 6.41 years in treated monkeys. In tissues showing notable signs of aging, metformin treatment demonstrated even more remarkable effects in slowing down the transcriptome clock. This was also the case for DNA methylation age. For example, DNA methylation age in the frontal lobe was rescued by 6.1 yrs with concomitant changes in the lung by 5.11 yrs, kidney cortex by 4.9 yrs, liver by 3.95 yrs and skin age by 2.65 yrs.

Significant improvements in the frontal lobe were seen in single-nucleus transcriptomic age (sn-transcriptAge) in brain cell types, such as the inhibitory neurons (5.59 yrs), excitatory neurons (5.45 yrs), microglia (6.86 yrs), oligodendrocytes (6.79 yrs), astrocytes (6.08 yrs), and oligodendrocyte progenitor cells (5.70 yrs). Age-related decline in morphogenesis, synapse assembly and increased markers of aging such as accumulation of p-Tau, SA-β-gal-positive cells, and pro-inflammatory markers (such as MMP9), were also attenuated following metformin treatment. Additionally, reduced amyloid-β accumulation, enhanced synaptic connectivity, and improved neuronal regeneration in the hippocampus. These findings suggest that metformin was neuroprotective against age-related damage. This is particularly significant for Alzheimer’s disease which is the most common type of dementia affecting 15% of patients aged 65 years and above. Indeed, metformin’s ability to decrease Tau protein levels, reduce amyloid-β accumulation and hippocampal senescence could also delay the onset of Alzheimer’s. In all departments of age-related disease further research is required.

**Fig. 1:**
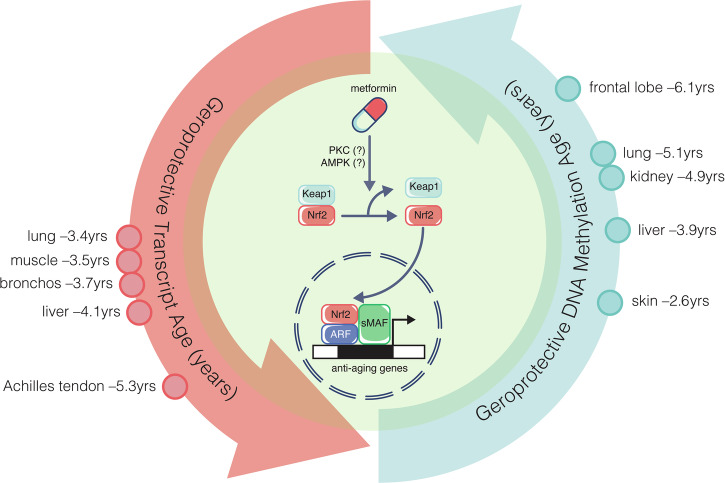
Metformin decelerates transcript and DNA methylation aging clocks. Comprehensive 40-month study shows geroprotective benefit of metformin in adult male non-human primates. One example of aging indicators slowed by metformin is illustrated with 6.1-year regression in brain aging, showing neuroprotection mediated by translocation and the activation of the Nrf2 transcription factor (Nuclear factor E2-related factor 2) when coupled with ARF (Auxin response factors) and sMAF (small musculoaponeurotic fibrosarcoma) to activate a program of antioxidant gene transcription

The results of a clinical trial (NCT01965756) showed modest benefit for mild cognitive impairment and early Alzheimer’s disease which may be attributed to the short period of metformin use at 8 weeks. This compares with an equivalent metformin administration of 10 human years in the NHP study.^1^ In yet another trial using metformin for 12 months, NCT00620191, that study found significant improvements in memory tests and cognitive functions in the patient treated group. These results were presumed to involve peripheral insulin resistance until clear links demonstrated metformin reduce oxidative stress through the nuclear factor erythroid-derived 2-like 2 (Nrf2).^3^ Oxidative stress has long been a key focus in aging research. The theory suggests that the accumulation of oxidative damage from reactive oxygen species (ROS) contributes to cellular dysfunction and aging. Over time, this damage can impair cellular processes, leading to functional declines in tissues and organs. Nrf2 is a transcription factor that is activated by oxidative stress. Under physiological conditions, Nrf2 is bound by Kelch-like ECH-associated protein 1 (Keap1) in the cytoplasm which inactivates it. During oxidative stress, Keap1 residues C273 and C288 undergo conformational change that destabilizes the Nrf2-Keap1 complex. Consequently, Nrf2 accumulates in the nucleus, to form a heterodimer with small musculoaponeurotic fibrosarcoma proteins and binds to the antioxidant response element, to activate the expression of cytoprotective genes such as HO-1, NQO-1, SOD3, GPX2, and GPX1, encoding antioxidant proteins (Fig. 1).

Furthermore, we also appreciate the loss of H3K9me3 as a recognized marker of aging that is associated with premature aging syndromes, such as Werner syndrome and Hutchinson-Gilford progeria syndrome.^4^ While the authors showed metformin administration restored H3K9me3 levels in monkeys, we couldn’t find clinical trials that have evaluated metformin treatment with premature aging syndromes. This could open the door for future research programs that comprise metformin’s action and efficacy in patients with congenital aging disorders.

While there are more than 20 clinical trials registered to study the influence of metformin as a geroprotective agent, an ongoing trial in Xuanwu Hospital, NCT06459310, registered by the same study authors investigates the efficacy of oral metformin (1000 mg) in middle-aged and elderly males. While the rationale behind the study design is understandable, there is growing recognition the science also address sex-specific effects. Females show higher incidence of dementia and cognitive decline than males.^5^ Furthermore, the purpose of the Targeting Aging with Metformin (TAME) Trial will examine the impact of drug intervention of multiple age-related conditions. The studies will be conducted by the NIH-funded Geroscience Network and will involve more than 3,000 individuals between the ages of 65–79 yrs and remains squarely focussed on metformin given its safety and low cost.

The age-old adage about rushing in where angels fear to tread applies with peculiar force to anyone who attempts to define the biology of aging. Now is the time for age-related metformin research. Our understanding of geroprotection are also converging because these stories are as old and as new as, cell cycle and transcriptional signaling. The science of these stories won’t be stopped. In much the same way, that current research and future clinical trials show promise for interventional drugs like metformin as a key to unlocking longevity, aging and neuroprotection.
